# Comparison of the burden of illness for adults with ADHD across seven countries: a qualitative study

**DOI:** 10.1186/1477-7525-10-47

**Published:** 2012-05-14

**Authors:** Meryl Brod, Betsy Pohlman, Robert Lasser, Paul Hodgkins

**Affiliations:** 1 The Brod Group, 219 Julia Ave., Mill Valley, CA, 94941, USA; 2 Shire Pharmaceuticals, 725 Chesterbrook Blvd., Wayne, PA, 19087, USA

**Keywords:** Attention deficit hyperactivity disorder, Quality of life, Patient reported outcomes, Cross-cultural comparison

## Abstract

**Background:**

The purpose of this study was to expand the understanding of the burden of illness experienced by adults with Attention Deficit-Hyperactivity Disorder (ADHD) living in different countries and treated through different health care systems.

**Methods:**

Fourteen focus groups and five telephone interviews were conducted in seven countries in North America and Europe, comprised of adults who had received a diagnosis of ADHD. The countries included Canada, France, Germany, Italy, The Netherlands, United Kingdom, and United States (two focus groups in each country). There were 108 participants. The focus groups were designed to elicit narratives of the experience of ADHD in key domains of symptoms, daily life, and social relationships. Consonant with grounded theory, the transcripts were analyzed using descriptive coding and then themed into larger domains.

**Results:**

Participants’ statements regarding the presentation of symptoms, childhood experience, impact of ADHD across the life course, addictive and risk-taking behavior, work and productivity, finances, relationships and psychological health impacts were similarly themed across all seven countries. These similarities were expressed through the domains of symptom presentation, childhood experience, medication treatment issues, impacts in adult life and across the life cycle, addictive and risk-taking behavior, work and productivity, finances, psychological and social impacts.

**Conclusions:**

These data suggest that symptoms associated with adult ADHD affect individuals similarly in different countries and that the relevance of the diagnostic category for adults is not necessarily limited to certain countries and sociocultural milieus.

## Background

Worldwide, adults are increasingly diagnosed with ADHD. Typically seen as a problem of childhood, and once thought to be something that people outgrow, clinical and epidemiological research have identified ADHD as a persistent condition in adulthood for at least a large minority [1-4]. In children, ADHD is a commonly diagnosed neuropsychiatric condition associated with hyperactivity, impulsivity, and inattention, and is estimated to occur in 3–7% of school-aged children [5]. In the United Kingdom, prevalence is estimated to be 3.62% in males and .85% in females [6].

In a meta-analysis of global prevalence in the general population, researchers estimated adult ADHD occurs at a rate of approximately 4% worldwide [7]. Persistence of ADHD in adulthood is strongly related to childhood symptom profile, including symptoms associated with the attentional plus impulsive-hyperactive type, symptom severity in childhood, comorbid major depressive disorder, high comorbidity of three or more child-adolescent disorders, paternal anxiety-mood disorder, and parental antisocial personality disorder [8]. Prospective studies evaluating the incidence of ADHD that persists into adulthood for those diagnosed with the condition in childhood range widely in their prevalence estimates, from 5% to 66% [9-13]. This wide range is largely attributed to methodological differences in the very few studies conducted and distinctions in the diagnostic criteria utilized [7,14]. However, ADHD among adults is understood to be a relatively common psychiatric condition with an estimated prevalence in the general population ranging between 2% and 6% [15]. The adult prevalence in the United States ranges from 2.9% to 4.4% [3,12,16,17]. Available estimates of adult ADHD in Europe range from 2.8% to 7.3% (France 7.3%; Germany 3.1%; Italy 2.8%; The Netherlands 5.0%) [18].

Importantly, the rate of recognition of ADHD contributes to the range of diagnosis estimates across regions and health systems. Recognition is, in turn, affected by many factors including challenges of diagnosis, varying definitions of functional impairments, adaptive strategies of individuals that may mask or minimize symptoms, problems of self-report and insight into childhood symptoms, and media representations that influence the perceptions of both doctor and patient [19]. These factors may contribute to stigma of the patient and his or her symptoms of ADHD. Additionally, health systems and insurance structures also impact diagnosis rates, reflecting national values and norms that create either obstacles or a pathway towards diagnosis. For example, focus group participants from Italy in this study often traveled to neighboring countries to receive both diagnosis and medications for ADHD. This reflected the view of the health system, and its practitioners, that ADHD was primarily a disease of childhood.

Many individuals are diagnosed with ADHD as adults without having been formally diagnosed in childhood. Currently, an adult diagnosis of ADHD requires a retrospective analysis of an individual’s childhood for behavioral aspects that meet the criteria of childhood ADHD. Researchers of ADHD in adulthood argue that the disorder does not occur spontaneously in adulthood [2]. Although there is some continuity of core ADHD characteristics, the manifestation of symptoms varies in a developmental manner. For example, whereas children with ADHD may experience difficulties across all core symptom areas (inattention, hyperactivity, impulsivity), adults with ADHD are thought more likely to have inattentive symptoms than hyperactive or impulsive symptoms [17,20].

Evidence suggests that ADHD symptoms continue to impair functioning, well-being, and health-related quality of life in adulthood [21-24]. Adults with ADHD often experience multiple life difficulties, including depression or anxiety, anti-social behaviors, lowered socio-economic status due to work-related issues, and substance abuse [2,4,11,25,26]. Adults with ADHD are at risk for social skill deficiencies due to difficulties in affect recognition [27] and anger expression [28]. Women with ADHD report more depressive symptoms, greater anxiety and stress, and lower self-esteem than women without ADHD [29,30]. In addition to the effect of ADHD on the individual, the societal impact of adult ADHD is significant as adults with ADHD commit more antisocial acts [25], are arrested more often [25], and are at increased risk for motor vehicle accidents than those without ADHD [28]. ADHD is relatively common in the workplace globally. As demonstrated in a ten-country study, approximately 3.5% of working adults have ADHD with related impacts on their work productivity [18]. From an economic perspective, healthcare costs are greater for adults with ADHD compared with those without ADHD [31]. Using a cost of illness framework, annual costs range between $12,005 and $17,458 per individual, with a total annual societal cost in childhood and adolescence of approximately $42.5 billion dollars (2005 estimates) [32]. Social stigma is also associated with behaviors exhibited by those with ADHD, compounding lifelong social suffering [33]. Thus, ADHD, as currently understood, imparts substantial morbidity on those with the disorder.

Over the past few decades, prevalence data have suggested that the United States incurs higher rates of diagnosis for ADHD generally, and this has catalyzed concern that ADHD might be an explicitly “American condition”, interpreted through specific socio-cultural structures and expectations for individuals. As researchers have noted, this may be due to the fact that research of ADHD has centered in the United States, and that there are discrepancies in the use of the diagnostic criteria [7]. The aim of this study was to qualitatively understand the ADHD experience in different countries of North America and Europe, with a focus on the similarities of its burdens across countries and health systems.

## Methods

This analysis was derived from fourteen focus groups and five one-on-one telephone interviews held in seven countries: Canada, France, Germany, Italy, The Netherlands, United Kingdom, and United States (two focus groups in each country). The five telephone interviews were conducted in Italy in order to balance the sample size with that of the other countries. Adults with a medical diagnosis of ADHD were recruited for each group either through local clinical experts or ADHD advocacy groups. Participants were compensated for their time spent attending the group. The study was approved by an ethics committee.

The focus groups were semi-structured and conversational, and constructed with an emphasis on the burden of illness for the participants. The focus group script was developed based on a literature review, feedback from expert clinicians from each country included in the study, and prior experience of the authors with this population. The guide was designed to elicit commentary in several areas, including how the participants were diagnosed, their childhood experience of ADHD, and daily life, social, psychological, and physical functioning. All focus groups in English speaking countries were conducted by the first author, an experienced group facilitator. In non-English speaking countries, the groups were conducted by native speakers who were experienced facilitators and trained by the first author on the focus and intent of the discussion guide. Transcripts were translated into English and transcribed by different transcribers, and then coded thematically by one analyst (the second author). A preliminary code list was derived from the focus group script, and codes were added to the list as new themes emerged during the review of each transcript. Transcripts were reviewed and coded at least three times. First, they were reviewed to assess basic content and the emergence of new themes. This was followed by a second in-depth coding session. Finally, they were reviewed a third time for accuracy and consistency in the coding process. Additional review of transcripts occurred when a new code was added, to insure that all incidences of that theme were captured during the coding process. This method is especially strong for revealing similarities across transcripts; however, the regular review and re-review of transcripts also potentially highlights differences between them. Throughout these comparisons, an effort was made to look for differences by country.

When appropriate, codes were merged to avoid redundancy and/or split to reveal more specificity in the themes discussed by participants. Codes were then grouped into domains. The coding process was undertaken by one individual (the second author), in consult with the first author. The end result was a full coding schema that was descriptive of the collective experience of adult ADHD, spanning from physical symptoms to participants’ psychological and social responses to them. Representative sample comments are provided following each domain summary to support the qualitative findings. This descriptive coding method is consistent with grounded theory and other thematic analysis methodologies [34]. These participant commentaries were chosen for 1.) descriptive quality and 2.) central focus on the theme in question. They do not reflect any preferences for specific treatments or medications.

## Results

A total of 103 focus group participants, 52 men and 51 women, participated in the 14 focus groups. The average size of a group was 7 participants, with a range of 3 (one of the focus groups in Italy) to 9 participants. In addition, five telephone interviews were conducted in Italy in order to include more participants from that country. This brought the total number of participants to 108, representing 51 women and 57 men. The mean age of patients was 36 years, and the mean length of time since the diagnosis of ADHD was 8 years (range 0 to 46 years). Thirty-two percent of the study sample was diagnosed with ADHD in childhood. The self-reported lifetime average number of ADHD medications taken for the sample was 1.7, with 55% currently undergoing treatment with a prescribed ADHD medication. As expected in an ADHD population, 42% of the subjects reported that they also had other mental health diagnoses besides ADHD.

See Table 1 for the full sample description.

**Table 1:** Demographics

**Sample Description (N = 108)**		
**Sex, n (%)**	Female	51 (47.2)
	Male	57 (52.8)
**Marital status, n (%)**	Single	50 (46.3)
	Married/partnered	41 (38.0)
	Divorced	15 (13.9)
	Widowed	1 (0.9)
	No response	1 (0.9)
Ethnicity, n (%) (n = 65)*	Caucasian	48 (73.9)
	African Caribbean	2 (3.1)
	Latino/Hispanic/Mexican American	2 (3.1)
	Asian/Pacific Islander	1 (1.5)
	Native American/Alaskan Native	1 (1.5)
	Other/Mixed racial background	9 (13.8)
	Declined to answer	2 (3.1)
**Age(years), mean [range]**		36 [18–62]
**Employment status, n (%)**	Full-time paid position	46 (42.6)
	Part-time paid position	16 (14.8)
	Not currently working for pay/retired	25 (23.2)
	Student	20 (18.5)
	No response	1 (0.9)
**Education, n (%)**	Less than secondary school	10 (9.3)
	Completed secondary school, technical school/ equivalency diploma	44 (40.7)
	College degree	39 (36.1)
	Graduate degree	14 (13.0)
	No response	1 (0.9)
**Income (USD), n (%)**^**†**^	Less than $20,000	24 (22.2)
	$20,000-$59,999	49 (45.4)
	$60,000-$99,999	14 (13.0)
	$100,000+	6 (5.5)
	No response	15 (13.9)
**ADHD Characteristics of Sample (N = 108)**		
**Age diagnosed with ADHD(years), mean [range] (n = 101)**^**‡**^		28 [4–59]
**Age first ADHD medication taken(years), mean [range] (n = 98)**		29 [4–59]
	Medication taken	98
	Medication never taken	3
	Respondent did not know age	2
	No response	5
**Currently taking ADHD medication, n (%)**	Yes	58 (53.7)
	No	48 (44.4)
	No response	2 (1.9)
**Number of medications used to treat ADHD, mean [range] (n = 106)**		1.7 [0–6]
**Mental health condition**	Yes	45 (41.7)
	No	58 (53.7)
	No response	5 (4.6)

*Question not asked on survey in France, Italy, and Germany.

^†^1 € (Euro) = 1.35117 US dollar ($); 1 British pound (£) = 1.53870 US dollar ($); 1 Canadian dollar (C$) = 0.98629 US dollar ($). Source: MSN Money, 4/16/2010.

^‡^No response (n = 7).

As noted earlier, the focus group script emphasized the burden of illness for ADHD. As a result, participants talked about dilemmas of life affected by ADHD. They also spoke of positive aspects of life with ADHD, although to a lesser degree because of the emphasis on the burden of illness. Their comments were grouped analytically into four domains: ADHD Medical History (diagnosis, symptoms and medication use), Life Experience, Daily Life Impacts of ADHD and Psychological and Social Impacts of ADHD. Each broad domain contained several subdomains or themes. In the sections that follow, we detail the burden of illness associated with ADHD. The number of respondents and percentage of sample that discussed the issues explored in a given domain is listed with the table heading.

### ADHD medical history

#### Diagnosis of ADHD

Over half of the participants in the focus groups and interviews (62 or 57%) reported that they were diagnosed with ADHD as adults (n = 108). Twenty-nine participants (27%) were diagnosed as children. There were 17 participants (16%) for whom the exact timing of their diagnosis was not revealed by them in the focus groups. Participants from all seven countries described diagnosis of ADHD in adulthood as a process involving multiple attempts to locate the cause of self-perceived problems. These attempts included misdiagnosis and self-diagnosis based either upon public media or encountering others who had the diagnosis of ADHD, sometimes in participants’ own children (Table 2).

**Table 2:** Diagnosis Experience (as adults or as children)

	**Diagnosis Experience (as adults)**
	**Approximately 62 participants reporting (57%)**^**1**^
**Canada**	*I was diagnosed with ADHD in the middle of March. I guess I had a bit of a nervous breakdown and I committed myself to [a hospital] for a month, and that’s when I got diagnosed.*
**France**	*Well it was their forum [the association Hyper Super] that helped me. I was looking for someone to help me with my daughter, and I recognized myself in a profile that was created on the forum. And they had a meeting for adult attention deficit disorder. When I saw the other attention deficit participants, I thought, that’s it, that’s me.*
**Germany**	*I started ADHD treatment 2 years ago and I’m very satisfied. I discovered that I had ADHD because of my child. I read about his condition and thought that I had the same symptoms.*
**Italy**	*I am 50, I am married, I have 4 sons, the first of them was diagnosed with ADHD 8 years ago. […] Finally I realized that, being a genetic disease, I had it too. 2 years ago I was diagnosed with ADHD by a child psychiatrist because I didn’t find anyone else in my area. […] I re-lived all my life and I realized what lacked, what I lost with this disease and what I coped with. I’ve been taking Ritalin for 2 years. I didn’t find it in Italy because it cannot be taken if you are an adult, but since my son took it, I started to use it.*
**The Netherlands**	*I was 48 years old, and had been thinking I had it since I was 42 because I had two images walking around the house and I had seen a program on ADHD in adults on television, recognized it and thought: “I’m not the only one”.*
**United Kingdom**	*I’ve only recently been diagnosed with ADHD in the last year […] I suppose I’ve always been different from other people, I’ve always been in a lot of trouble throughout my life, I suppose it kind of explains some of it now.*
**United States**	*I had a doctor try to tell me I was bipolar and put me on a bunch of medication for bipolar and I ended up crazy, like shaking, crazy. […] Every time I went in I had a new side effect so they would put me on another medication. They had me up to eight […] The psychiatrist basically said, “Let’s take the medication all away.” Every dose I went down I felt amazingly better.*
	**Diagnosis Experience (as children)**
	**Approximately 29 participants reporting (27%)**
**Canada**	*I’m 41 and it was first noticed when I was in Grade 2. I was just always getting into trouble and alienated from my class. My mom was a nurse and she took me to the doctor and they did a bunch of tests and it came out that I was ADHD. All I remember is the term hyperactive disorder. But my mom didn’t want me to go on the pills. She thought that she could just kind of parent it out of me, which didn’t have the greatest effect on my life.*
**France**	*I discovered my ADHD when I was 3 years old. I took Ritalin, Concerta. I took them for a year and a half, and stopped because I had too many side effects. I had the impression that I got nothing out of the treatments. That’s it.*
**Germany**	*ADHD is something that I’ve known of many, many years ago. It was actually in first grade because I had difficulties with writing and learning how to write with fine motorics and there was also psychological treatment. I’m from Chemnitz in East Germany and at the time they had just a pilot project at the technical university in Chemnitz. I was there as a patient for 2 years, then I took Ritalin but after 9 years it wasn’t paid for anymore and that’s how I got hooked up on methylphenidate and I’ve been taking it for the last 3 years.*
**Italy**	*I was diagnosed with ADHD at 16. I started to take drugs at 16, I’ve not been taking drugs for 1 year now. Besides Ritalin, I also took Concerta. I started with Ritalin and I took it for 3 years but I stopped it because I didn’t need it any longer. I took Concerta especially when I went school. I alternated Ritalin with Concerta during the school years and also the 2 years after school. I stopped to take it for the same reason as Ritalin’s.*
**The Netherlands**	*For me it has been clear ever since I was little. But back then the doctors had just discovered ADHD so everybody had it. They thought everybody who had difficulties concentrating or was unmanageable had ADHD. ****[LEADER: But for you it was because you had problems concentrating?]****Yes, concentrating, being super active, the whole package. It was a bit of a tug-of-war with the GP about what had to happen. Ritalin was brand new back then and came from America. So it never came to that.*
**United Kingdom**	*I’m 22 and I got diagnosed when I was, I think I was about 16, 17 at the end of school. I always sort of had trouble at school, I got in trouble a lot. I sort of knew that there was something wrong myself and one my teachers suggested it because my mum didn’t, still to this day doesn’t think there’s anything wrong with me. So, I went to the, because I was old enough to go to like the adult psychologist at that time, so I went myself and then they diagnosed me and I took Ritalin but it made me sick, so I couldn’t have that. I took Concerta and it did work for the first few weeks but it sort of makes me like a zombie so I sort of stopped taking it. So, I haven’t taken anything for about a year now.*
**United States**	*Actually, I was diagnosed with this when I was 10 years old and a psychiatrist prescribed Dexedrine. But I have a mother, and she didn’t like it. This was a long time ago when people went home for lunch from school. She didn’t like that I wasn’t eating the lunch so she decided it would be better if I didn’t take the Dexedrine. It kind of crippled my relationship with her for a while.*

^1^All participant numbers in all the tables presented here are approximations only due to variations in transcription styles and the inability to consistently discern individual speakers.

### Symptoms of ADHD

#### ADHD symptom agreement

Participants from all focus groups demonstrated an agreement on the primary symptoms associated with ADHD: hyperactivity, impulsivity, inattention, and impairing disorganization. Tables 3,4,5 and 6 offer representative statements for each symptom type, with approximate participation. Of special note are the numbers of participant reports of continued hyperactivity and impulsivity as symptoms of ADHD into adulthood, as these symptoms are thought to decrease with age [17,20]. However, approximately 60 (56%) participants reported hyperactivity and 67 (62%) reported impulsivity as ADHD symptoms they experience. This compares to 69 (64%) participant reports of inattentiveness and 47 (44%) participant reports of disorganization, symptoms thought to be most concerning for adults with ADHD (Tables 3,4,5 and 6).

**Table 3:** Hyperactive Symptoms

	**Hyperactive**
	**Approximately 60 participants reporting (56%)**
**Canada**	*Hyperactivity, yes, because I used to go to bars and dance all night, and I’d work all day, go to bed at 4 o’clock in the morning whether I took drugs [for treating ADHD] or not. I couldn’t stay in place.*
**France**	*When I’m in a meeting, I have to move. One problem I had in school was that I’d just get up and walk out.*
**Germany**	*We had to sit for 7 hours and that was a nightmare. I do everything rather than to sit down quietly. You sit there and get unrestful, how can you cope with it? I find it very hard.*
**Italy**	*My hyperactivity consists in not being able to do one thing at a time, I always make two things contemporaneously. Being sat down in this chair for 2 hours is a torture for me. […] I continuously change position.*
**The Netherlands**	*I know that I tend to exhaust myself. […] Sometimes you have 20 or 30 ideas a day, and they will keep you busy for the rest of the day. In the meantime there are also some you try to get done physically. […] And then I’m useless for the next 2 weeks.*
**United Kingdom**	*I used to go dancing like five times a week and mum always made sure I had things to do, but I could come home from like a theme park and I’d be like what are we doing now? I’m bored. […] That isn’t normal. […] I was constantly like what are we going to do now?*
**United States**	*I have to be doing something at all times. […] I always have something. That’s why I can’t sit and watch TV. I always have something I have to do.*

**Table 4:** Inattentive Symptoms

	**Inattentive**
	**Approximately 69 participants reporting (64%)**
**Canada**	*I just run around in circles for nothing at home, you know, like walk around, get lost, I forget what I was doing, come back, start something else […] it’s not that I’m Alzheimer’s, I just forget.*
**France**	*My problem is that when somebody has been talking for five or ten minutes, I kind of fade out, I just answer automatically, yes, yes.*
**Germany**	*I said something to people when I wasn’t aware I was saying it to them but I thought I was only thinking about it. […] It happens to me quite often lately. If I delegate something to others I think I have said it to them but I actually haven’t done it.*
**Italy**	*Moreover, every single noise bothers me: even if the doors are closed, I can hear the noise made by the washing-machine coming from the 3*^*rd*^*floor, voices of the people who are talking. They really bother me.*
**The Netherlands**	*I have finished my education in 4 years, while the Intermediate Vocational Education says it takes 2 years. That’s because of my inability to concentrate.*
**United Kingdom**	*Say if I get a book and I really enjoy reading it, half way through I’m done. There’s no energy in me to even bother reading the rest of it because I just can’t concentrate. I just do something else and don’t really focus on one thing.*
**United States**	*I’m really conscious of the fact that I’m squiggly or whatever else I’m doing that is moving me around that or the fact that I don’t know what to say or I can’t pay attention. Did I say something really stupid?*

**Table 5:** Impulsive Symptoms

	**Impulsive**
	**Approximately 67 participants reporting (62%)**
**Canada**	*Sometimes I start talking too much, and I feel ridiculous. […] But sometimes I start talking weird and I’m saying, “Gee, what am I talking about?” Like I’m talking about Godzilla all of a sudden. You know, it’s like super-weird.*
**France**	*When I think a teacher is wrong, I don’t have the wisdom, as my best friend says, to shut my trap.*
**Germany**	*Yes, exactly that’s the same with me. Yes, I’ve got this impulse and it needs to get out and you make yourself very vulnerable to many people.*
**Italy**	*The biggest problem I had and that heavily penalized me in my social life was my impulsiveness: I always fidgeted, I didn’t have self-control because I was not aware. […] So, my main symptom was impulsiveness.*
**The Netherlands**	*When I have something in my mind that I want, not necessarily impulse purchases, but when I want to do something that seems like fun. Then I really want it and it has to happen right away.*
**United Kingdom**	*I’m a classic put my foot in your mouth personality. […] It just is a bit of a nightmare, things like that. It’s always a constant battle to think about what I say and how he’s going to hear what I say. […] I think I’m a bit too honest.*
**United States**	*But say I’ll lock the back of somebody’s car while they’re at a stoplight or something, or I’ll pull practical jokes for no reason. And it’s not even that it amuses me—it’s just that the thought goes through my head and my body carries it out.*

**Table 6:** Disorganized Symptoms

	**Disorganized**
	**Approximately 48 participants reporting (44%)**
**Canada**	*It’s a real struggle for me to keep a certain order. […] So all of a sudden I’m really functional and everything, but then all of a sudden I’ll become dysfunctional and I don’t know why, and it’s like chaos, and I feel stuck in that.*
**France**	*I’m always feeling behind on things I was supposed to have done. It’s something I can’t get over. Disorganization is something I can’t conquer.*
**Germany**	*On the downside I see ever more chaos spreading around myself. […] I mean if you look in my room I’ve always got an island in my room that is like a stack that will never vanish.*
**Italy**	*I always jot down on papers and I accumulate them in so many piles of papers. After a ring, sometimes I just write the name or the phone number down and I end up forgetting who they are. Sometimes I lose my house or car keys. They’re everywhere because when I come back home, I put my keys in one place, the wallet in another.*
**The Netherlands**	*I had that feeling the other day at home. There were so many thing laying on the floor from doing jobs, that I didn’t know where to start with cleaning up. It took 3 days and then you just start somewhere.*
**United Kingdom**	*Yes, me too. My car’s disgusting. […] I’ve got everything, clothing, food, drink, everything in there. It’s like my little house. I don’t know why it gets like that, I just leave things in there and then forget to take them out, or can’t be bothered.*
**United States**	*I’m so disorganized. […] People know when they come to my house, my sister, people have come to my house and I’ll say, “Don’t touch that pile. Don’t touch that pile. I know exactly what’s in them so don’t touch them.”*

### Medication use and adherence

Among the participants who chose to take medication for their ADHD, their comparisons between pre- and post-medication use generally favored the use of medication to treat symptoms of ADHD. However, as noted earlier in the descriptions of the focus groups, many participants did not take medication for reasons ranging from an opposition in general to taking medication to a dislike of either its effects or side effects (Table 7).

**Table 7:** Differences On/Off Treatment

	**Differences On/Off Treatment**
	**Approximately 56 participants reported medication use (52%); 51 (91%) reported positive effects of medications**
**Canada**	*Now with medication I’m organized, I can do my job. Like, I feel so organized, files and stuff, and I take notes, and I underline things and, you know, highlight with different colors, and I feel so organized.*
**France**	*Before I took the treatment I used to do several things at the same time. Now, with treatment, I cannot do many things at once. It makes me concentrate on one thing at a time. Also I used to have very strong emotions before but the treatment has stopped that.*
**Germany**	*Since I’m in treatment I’m so much more relaxed these days, I can listen now, I don’t have the necessity to keep talking all the time. […] I don’t need to interrupt people anymore. I’m just way more relaxed about the relationship. […] I have to say with the medications I have become a more sociable person. I don’t take myself as seriously anymore.*
**Italy**	*When you don’t take it, concentration lacks, concentration is the thing that changes most. Being concentrated means not making mistakes.*
**The Netherlands**	*It is a difference like day and night [taking medication]. Actually my ADHD hardly bothers me anymore, except for that I have to be constantly triggered at work and my hobbies always have to be dangerous and go rough.*
**United Kingdom**	*It was like the first time I took medication was the first time I’d ever watched a film the whole way through without moving, and I read my first book ever but I didn’t like my friends that I’d been friends with for years, and I’d be upset about my lifestyle from before, now I started realizing the dangers I’d put myself in, the fear.*
**United States**	*I used to be, “The more the merrier.” I had 20 friends. When I wasn’t on medication, I could manage the 20 friends. On medication, I avoid it. It’s weird.*

Participants reported many side effects from the medications typically prescribed for ADHD across all focus groups, and concerns about side effects often led to discontinuation of medication use (Table 8). Nearly all individuals with a diagnosis of ADHD had tried medication at some point in their lives (either as children or as adults), but their continuation of the medication depended largely upon their perceptions of efficacy. Some were continuing to take medication, others were ambivalent and might try it again, and a smaller number were convinced that medication would never provide a solution to what ailed them

**Table 8:** Side Effects

	**Side Effects**
	**Approximately 59 participants reported side effects (55%); 51 (86%) reported negative side effects that affected whether or not they continued with the medication**
**Canada**	*For one thing, one of them made me really nauseous, really sick. […] I think it was a twelve-hour medication. And within a half-hour of me taking it I just felt like I wanted to throw up. I felt so drawn into things and I was really alert and I felt very sickly. But I was on it for six months, and eventually I begged my parents to take me off it.*
**France**	*I was taking Ritalin. I stopped because I felt too drowsy. I think it wasn’t good for my health.*
**Germany**	*Yes, there is one thing that I stopped because I got extreme eczema, extreme rashes on my skin but it took me a long time to find out that it was a connection between the medication and these skin rashes but apart from that I couldn’t speak of any side effects. It is like real crevices and really horrid in my face and all over my skin.*
**Italy**	*So many. Even insomnia, every night I had frozen arms and legs, I had nightmares, but skin problems (especially with mosquitos) were the worst of all problems (I never had before and after the therapy) and most of all respiratory problems (I didn’t breathe well, I yawned every moment) and at the same time I had no benefits. Only negative effects. […] Since then I have not taken anything.*
**The Netherlands**	*You have the calmness, mentally I felt good because I had rest. But physically, I lost weight, I didn’t eat anymore. […] And those chest pains, I had those too. Chest pains.*
**United Kingdom**	*I took Ritalin but it made me sick, so I couldn’t have that. I took Concerta and it did work for the first few weeks but it sort of makes me like a zombie so I sort of stopped taking it.*
**United States**	*I quit just because of the jitteriness…*

Additionally, participants reported taking their medication in an as-needed rather than a routinized, prescribed manner. Some participants reported the use of alcohol and other drugs explicitly as a type of self-medication and as a self-care effort to quell their ADHD symptoms and find normalcy, calm, focus, and self-respect (Table 9).

**Table 9:** Compliance or Adherence

	**Compliance or Adherence**
	**Approximately 39 participants reporting (36%)**
**Canada**	*For me, I feel like being myself, and if I don’t take it on the weekend it’ll help me, because I don’t want to take it for a lifetime, so I want to be able to be myself without it so that there can be this transition. I want to have these good habits and develop good habits, and I don’t see such a difference now between the weekend and the week days.*
**France**	*You could say I have no memory. I forget to take my medication. […] I didn’t take it during the holidays because I didn’t really want to, I wanted to relax and didn’t need to manage as much. […] Yeah when I have had enough of it. Had enough of the effects it brings.*
**Germany**	*Yes, I use them very deliberately, very targeted. […] When I know I’m hitting a wall again and if I see that wall building up in front of me for example like I can’t even get up from my chair anymore, can’t actually clear away anymore because I just can’t do it at that moment or because I can’t get started with something […] then I know that I’m in a situation when I have to take half a pill. Because it is just something like a kick starter that gets me going. But I don’t like it if I’m taking something that’s constantly feeding on me the whole day. […] Actually I adjust it to my feelings every day.*
**Italy**	*I take the drug discontinuously (for example when I have to study or to drive) because sometimes I don’t need it. […] Actually I take it most of the time because I’m a student, but if I go on holiday I don’t need it. I only take it to do certain things, if other things don’t cause tiredness I don’t take it.*
**The Netherlands**	*And if I have forgotten my pill, sometimes on purpose, I feel very sad or happy the next day. But it’s good feeling sad for a day sometimes. Then I can feel again. When I take my medication every day, every day’s the same.*
**United Kingdom**	*I find it difficult to stick to routines, so I take them three times a day as I do most of the time, but if I get up early, then that usually throws my schedule, but I do take them all the time. I have at least two of the doses, but I try to go to three. I try to take them as consistently as possible.*
**United States**	*I don’t take them exactly at a certain time. I just take them on an as needed basis. If I have a task that I have to get accomplished or sometimes after lunch if I ate too much and I feel a little dicey, I’ll take some meds so I can actually get something accomplished in the afternoon hours.*

### Life course experience

#### Childhood experience

Participants from all seven countries noted that they had problems in school with learning and in social interactions with classmates and teachers when they were young. Narratives concerning school-age experiences include being bullied, struggling with depression, feeling restless, feeling misunderstood, and early-age drug and alcohol abuse (Table 10).

**Table 10:** Troubled Childhood Experiences

	**Troubled Childhood Experiences**
	**Approximately 55 participants reporting (51%)**
**Canada**	*My childhood was terrible and my teenage years were terrible, and now I’m feeling much, much better. I was walking on the sidewalk feeling terrible about myself, lost, and no self-esteem. […] I know I was in pain, I was suffering. It’s like another life, as if I was reborn. I remember very vaguely being just another person.*
**France**	*No I wouldn’t say that I felt isolated or alone but I had a problem with memory. For example, at school I would forget my book and would be told to bring it next day and I would lose things.*
**Germany**	*When I was kid I was extremely restless, one of these typical restless kids when I was 7 or 8 and then nothing much happened. Later on I became a more quiet person, slightly depressed sad kid and teenager.*
**Italy**	*I didn’t have any friend until the first year of the high school, when I started to take the drug. I had no friends. Before I took the drug, they pulled my legs because of my behaviors. My younger brother was always beaten because he was the weirdest.*
**The Netherlands**	*As a young child you of course are in the age in which you develop yourself and try out one another, you fight every once in a while, it’s all part of it. Kids just do that… but ADHD’ers keep on going and can’t stop with a joke for instance, which automatically puts you outside the group. It’s frustrating because you can’t calm yourself down, that’s very annoying.*
**United Kingdom**	*But I was depressed as a child, depressed, lonely, bullied, and teased and everything. My parents hated me, my sisters hated me. They were always tormenting me at school, you know? I didn’t have many friends.*
**United States**	*I remember having problems if there was any noise or movement especially if a teacher was talking to another student when we were supposed to be doing our work. I couldn’t concentrate or maintain focus. I would read the same paragraph over and over and over again and it would not sink in. […] As a kid, people would call me an adrenaline junky.*

#### Impacts beyond childhood

Early life experiences in school and at home were characterized by participants as having an enduring quality on their lives. Rather than discrete events, general difficulty in school(s) impacted their subsequent life. Many adults with ADHD feel that had they been diagnosed and/or treated differently in life, they would have completed school more successfully. This included acquiring more advanced degrees, affecting their career success and overall life experience (Table 11).

**Table 11:** Negative Impact of ADHD on Life Course

	**Negative Impact of ADHD on Life Course**
	**Approximately 69 participants reporting (64%)**
**Canada**	*I remember the dilemma for a long time was that I had no self-esteem, comparing myself with other “normal” people, and maybe I would have succeeded professionally much better if I had been able to concentrate longer. But I had such a short attention span I would change careers for nothing.*
**France**	*I missed out on my studies. I didn’t get anything out of school, so I didn’t realize my potential. […] If I’d really been able to work at my full potential, I could have ended up with a good diploma.*
**Germany**	*I think if it had not been diagnosed early in my life I would have taken a different course. I couldn’t even write early in school. I worked as a car mechanic, I worked in a woodwork shop and I did everything that is physically demanding.*
**Italy**	*So if you had known your condition before, you would have chosen a job that fits you and you wouldn’t have chosen for convenience, for having more job opportunities or for settling down. It is already hard to concentrate, and if we do a work we don’t love or that does not correspond to our talents, failure is around the corner.*
**The Netherlands**	*My parents would have understood why I’m sometimes acting strange, why I’m sometimes this active and feeling like doing nothing the next time, and why I’m cranky to them. Maybe I would have been able to have a more normal contact with them, even with my mood swings, and I would have been able to finish my education more easily.*
**United Kingdom**	*I went to four different colleges. I dropped out three different times. I don’t know how I made it through. It was just sheer will that I did it, but just the emotional turmoil I went through was just horrendous. […] I think if I had known earlier, people would’ve been nurturing about it and my parents would’ve advocated for me at school. I think I would’ve had better outcomes.*
**United States**	*I'm sure I would have made better grades because I lose my concentration and if I would have had medicine, I’m sure I would have done a lot better in school and I probably would have gone to college.*

#### Addiction and risk-taking behavior

Dangerous or risky activities were described by participants in all focus groups, and included past and present misuse and abuse of alcohol and drugs, eating disorders, an altered ability to assess risk, and an enjoyment of extremes (sports, challenges, or job types). Driving cars aggressively and recklessly, often accompanied by anger directed towards other drivers was commonly mentioned, consistent with the ADHD profile (Tables 12 and 13).

**Table 12:** Addiction and/or Substance Abuse

	**Addiction and/or Substance Abuse**
	**Approximately 42 participants reporting (39%)**
**Canada**	*I guess it developed in high school, and then of course I got into drugs, but I think it’s because I wasn’t able to concentrate. […] And that lasted ’til the end of my 40s, drugs, mental health, all that happened at once, you know? I didn’t want to be in contact with the present reality. And I got many diagnoses for other things before they diagnosed the ADHD.*
**France**	*You’re too ready to put yourself in danger. For me, putting myself in danger is a way to feel alive, to prove myself. […] You know, driving too fast, taking drugs, addictions, alcohol. It’s also a way of sending out an alert, maybe not consciously, but telling other people, “Something’s wrong with me.”*
**Germany**	*Yes, addiction is something that comes in all shapes. I must say that I’m quite addictive. I managed my addiction but now I’ve got other things: I bite fingernails for example, I ruined my fingers. I also had that experience that you spoke about, I lost weight I was down to 47 kg because I was running and not eating. Always these extremes, we don’t find a middle-way, we always just oscillate between the highs and the lows.*
**Italy**	*We cannot evaluate the danger.*
**The Netherlands**	*I have a subscription at the hospital for having so many accidents and fights, excessive alcohol intake, drugs.*
**United Kingdom**	*From my perspective I spent my youth, and most of my adulthood, up until the past couple of years, drinking anything I could get my hands on, and drugs but not that much, but I’ve tried everything except heroin. Of course, not surprisingly my favorite was cocaine because it made me feel normal again.*
**United States**	*I really start to feel like a little Tasmanian devil getting in my way all the time, like kind of self-sabotaging if I’ve got a deadline. […] It doesn’t matter what it is, what addiction it is. I haven’t struggled with drugs. I’ve had some issues with drinking. […] I feel like I’ve not had like one struggle, but I’ve had many that all kind of reflect that same idea of: what can I do to avoid something? And you can feel a little bit impulsive or compulsive, like you know you need to do one thing but I’m going to go on the Internet or get completely immersed in something different.*

**Table 13:** Risky Driving

	**Risky Driving**
	**Approximately 42 participants reporting (39%)**
**Canada**	*I’m a pretty aggressive driver. I get really impatient when cars are driving really slowly. I just feel like tailgating them. I’m really bad about that. But I try to calm down sometimes. I don’t know. I get really upset when people don’t use their signals. I just get really—it makes me so mad sometimes when I’m driving!*
**France**	*Also I find it hard to drive. I sometimes go through red lights and when I am asked why I did it, it is impossible to defend myself, I just did it.*
**Germany**	*Apart from that in my daily life—driving a car is also something, yes it is true if I’m inattentive I drive very fast and I even drive faster than my husband. […] But if I do drive I’d rather go quickly and fast and I want to get quickly from A to B. I mean bearing the traffic jam for example that’s something that really puts me on the edge.*
**Italy**	*My typical problem is speeding. […] I drive fast in order to arrive as soon as possible, because I’m always late. […] It’s a general characteristic.*
**The Netherlands**	*People who drive too slowly drive me crazy. […] I would prefer to drag them out of the car. When an old lady drives very slowly in front of me, I would like to take away her license. […] Or people who enjoy to drive alone in a traffic jam and create a gap of 40 meter. […] Then I like to honk and I am really angry.*
**United Kingdom**	*… but day to day stuff like driving, yes, maybe I do have a bit of road rage on a daily basis when I think about it. I do get annoyed but then I honestly don’t think that’s me, that’s other drivers and it frustrates me and stuff. Sometimes maybe I should because it could probably get me killed one day, but I don’t think about it at the time.*
**United States**	*I was in a near-fatal crash because I was changing the CD player. No one else was involved. This was when I was nineteen. […] And there was another time when I was driving and I wasn’t looking. […] Sometimes you’re like, oh, you’re just careless.*

### Daily life impacts of ADHD

#### Work and productivity

Work and career were topics of importance in all focus groups, and many individuals spoke of work as a troubled area of their life, one that required significant coping skills and supports in order to make it successful. Disorganization, forgetfulness, impulsive talking, problems with authority, and inattentiveness/distractibility were identified as obstacles in finding success in work. Participants in all focus groups spoke of difficulties in the daily activities of life, noting confusion, slowness, fatigue, inability to begin or prioritize, anxiety, and forgetfulness as obstacles to accomplishing tasks (Tables 14 and 15).

**Table 14:** Work Difficulties

	**Work Difficulties**
	**Approximately 79 participants reported work issues (73%); 69 (87%) noted difficulties with work**
**Canada**	*I had to quit school. I couldn’t follow. […] So I went to a public school where I just wanted to have fun, and I became a manual worker because I couldn’t do anything else.*
**France**	*I never spent more than 2 or 3 years in the same job because… I did things differently to other people. Another thing was that I sometimes had to mime, to pretend to be concentrating when I couldn’t.*
**Germany**	*It hinders your job performance. Often what people demand from you on the job, in your professional life, continuity, working hard, being realistic if you really have to work hard and be the supplier for your family that is a different story.*
**Italy**	*I am lucky because I have a secretary at work and my wife at home and they solve part of my problems. If I have a work due on next week, I postpone it one day, then two days, the last day I manage to finish it all. I am an architect. You always postpone.*
**The Netherlands**	*One day you are in a super condition, you see everything like you have 4 eyes and you’re doing very well. But the other day you will just sink in because everything takes so much energy, you have some kind of relapse. And when you are doing very well one day, people think you are capable of quite a lot, but the other day I show a lot less quality.*
**United Kingdom**	*I’m a medical student and I think ADHD affects me differently depending on the setting. If I’m on the wards it doesn’t affect me much because I’m on my feet and I’m going from patient to patient and it’s just constantly changing, so I don’t really have the opportunity to get distracted but if I’m sitting in on a clinic with a consultant and a patient, then my mind can wander because I’m just observing passively and I start to get distracted and look out the window.*
**United States**	*It’s hard to sit still in my own meetings. I squiggle. I don’t know what other word to use. You are sitting there with people who don’t and they look at you and I just don’t think I’m taken as professionally serious because I’m sitting around—we can all be dressed to the nines in our suits or in my case in heels and whatever but I’m a squirmer.*

**Table 15:** Problems with Productivity

	**Problems with Productivity**
	**Approximately 31 participants reporting (29%)**
**Canada**	*I can’t stand being late, but then I’m never on time. I was a waitress for 17 years, I’d get up too late, I’d get to work at the last minute, and I was functional. If I got there earlier I didn’t feel well, I wasn’t able to do my work. When I got there at the very last minute I was really productive.*
**France**	*You forget important appointments, important things you’re supposed to do. You’re not productive in society. You can’t really do something and come up with a result.*
**Germany**	*I always find it hard to get started with something. Every change of activity causes a problem to me and sometimes I have to lie down for 10 minutes because I’ve got this frightful dimness and tiredness before I can actually get started or I’m overpowered by times.*
**Italy**	*I have problems with memory, I cannot manage house chores. […] In the morning, I always have a lot of things to do, work in arrears, I start to do one thing, then another thing and I feel immediately tired, but I don’t finish anything and I haven’t done anything yet.*
**The Netherlands**	*I used to finish hardly anything, but not anymore. At a certain moment you just have to, otherwise the problems will simply pile up. You will sooner or later run into the things you haven’t finished and get the consequences back from it.*
**United Kingdom**	*I’ve got so much to do and I just look around and it could, I could sit there all day looking around thinking well where do I start? Then I get nothing done. It’s kind of up and down like that, but if I start, if something interests me and I start it, then boom, boom, boom, it’s done, but I have to almost like prepare myself.*
**United States**	*I have projects out the wazoo. I will tell you that I’m very creative and artistic but I never finish it. I never finished anything until I was on medication. Even now I have fifteen projects sitting there waiting to be finished. I notice when my dose is too strong that I focus way too much on one thing and never move on to the next. It’s like I can’t move on to the next.*

#### Finances

Financial management was an activity of significant trouble for participants in all focus groups. Problems with compulsive shopping or wasteful spending, in addition to the difficulties of money management (paying bills, setting priorities, knowing how much money they had), were noted in each focus group (Table 16).

**Table 16:** Problems with Finances and Spending

	**Problems with Finances and Spending**
	**Approximately 53 participants reporting (49%)**
**Canada**	*Not, I mean, a huge debt but yes. Once, you know, I didn’t have money for my credit cards and I had to ask my parents to pay off my credit cards because of this compulsive shopping, and it happened more than once. It’s not enormous, not like a house, but it was a lot of money.*
**France**	*I have a constant problem of overspending. I no longer have the right to go into overdraft. After a long period of unemployment I was on a government-assisted income, so I don’t have a choice. I have to be careful.*
**Germany**	*I don’t like doing my finances. That is difficult. […] I keep paying the fees and I often need to pay more, I get reminders because I don’t pay in time. I don’t pay my bills in time, so I pay additional money.*
**Italy**	*I could ask now “Who has paid more fines for not having paid lighting and water bills, car insurance on time?” because that’s what often happens.*
**The Netherlands**	*I am still chaotic and do not know what bills I am exactly paying. […] It is possible to do this together with someone else, but then I do not pay any attention and start thinking about smoking pot. It is just too hard and I know it. And it is a real mess.*
**United Kingdom**	*I’m just terrible with finances. I have this real emotional loathing of them, you know? It’s not just that I hate doing it; I also feel very upset, and when I see a bill and it’s a lot of money, I just go ballistic every time.*
**United States**	*I’ve given so much money to libraries because my books are late or I lose them. […] Not paying bills—I’m in some serious debt and it’s very hard to want to face that.*

### Psychological and social impacts of ADHD

#### Relationships

Irritability, inattention, impulsive talking, and forgetting contribute to misunderstandings in social interaction. Participants across all focus groups reported difficulty with a wide range of relationships, including those with parents, partners, friends, work colleagues, and acquaintances. Many reported that they have family members who also have ADHD, and some are partnered with people who have ADHD (Tables 17, 18, 19 and 20).

**Table 17:** Problems with Parents

	**Problems with Parents**
	**Approximately 27 participants reporting (25%) with 22 of these describing difficulties in relationships with parents (81%)**
**Canada**	*My mom was a nurse and she took me to the doctor and they did a bunch of tests and it came out that I was ADHD. All I remember is the term hyperactive disorder. But my mom didn’t want me to go on the pills. She thought that she could just kind of parent it out of me, which didn’t have the greatest effect on my life.*
**France**	*“Helping” is not the first word that comes to mind when I think about my mother. She pushes me, she makes me be more organized. She says, “If you don’t decide where your books are going to go, then I decide now. Your books are going to go on that shelf.”*
**Germany**	*I also come from an ADHD household. […] My parents are […] incapable of leading a decent and normal life and I think they are very messy because they simply can’t cope with it. They are compulsive in not throwing away and I really started cleaning at home at the age of five […] and I must say that I became a compulsive cleaner but always with that great fear if I was only to give way a tiny little touch of that then everything would break out in this huge chaos.*
**Italy**	*The most serious consequence of the fact that I have had ADHD since I was born is that my mother perceives me as a different person and came even to hate me. Because, in her opinion, I was not normal.*
**The Netherlands**	*My dad then says that I act so strange, but I have been doing that for half my life now. A little out of scope everywhere, at one time too active, at the other not feeling like anything, then feeling like everything again. It just shows.*
**United Kingdom**	*My schooling, I got thrown out. […] because they said I was badly behaved and, no one knew what was wrong with me, and when my mum took me to the doctors they actually said do you want some tablets? […] and she was like no! […] When my brother was born she thought there was something wrong with him so she took him to the doctors and she was like I’m sure there’s something wrong with my son and […] it was actually me.*
**United States**	*[My mother] decided it would be better if I didn’t take the Dexedrine. It kind of crippled my relationship with her for a while.*

**Table 18:** Challenges with Parenting

	**Challenges with Parenting**
	**Approximately 16 participants reporting (15%)**
**Canada**	*We’re running late to everything so it directly affects her [daughter], and then she’s embarrassed to walk into her class late. So I feel like I’m always letting her down. […] We miss the beginnings of everything. And I just feel like she is living with someone that has a disease.*
**France**	*After having treatment, I found my children again and was able to rebuild my relationships with them. I explained to them what had happened and why I was taking treatment and as they had witnessed my shortcomings first hand they recognized that it was an illness.*
**Germany**	*I regret it especially with the education of the children. Here it could have been helpful because there was a lot that could have been better or we could have done it better had we known that we had ADHD.*
**Italy**	*My elder child makes me suffer because he makes me re-live all the most difficult and embarrassing moments of my life because now it’s his turn. I can help him put these thing in perspective, I can orient him but I cannot live his life, I let him live it.*
**The Netherlands**	*When I’m at home I can be a real bully, I have no patience and I react fiercely to everything. I thought let’s try the medication and see if things can be handled better.*
**United Kingdom**	*I’ll have times with my son where everything’s going well for a while, then when my life goes downhill it affects my son really because he’s going back to his dad's house so that his dad get him to school on time and, just with his schooling it’s really where I’m not organized, like getting him to school on time. Some mornings I’ll be depressed, some mornings I don’t get up, I forget to put the alarm clock on. Then when he comes home from school I’ll forget that he’s coming home.*
**United States**	*I can’t even focus and hold a decent conversation with my kids unless I’m in the car and the radio is off.*

**Table 19:** Problems with Partner/Dating Relationships

	**Problems with Partner/Dating Relationships**
	**Approximately 64 participants reporting (59%) with 54 of these (84%) discussing tensions and challenges in partner/dating relationships**
**Canada**	*I have a wonderful husband, and he’s very patient. […] He finishes all the work for me.*
**France**	*[My partner] is an even bigger perfectionist than I am. He’s always got some idea or plan or scheme in his head. He’s not been diagnosed yet. I had a hard time convincing him to go to [a clinic]. He’s super-stubborn, but he’s the only person I’ve met with whom I don’t get bored. I had to find another hyper like me to better know myself.*
**Germany**	*When I got married, when my life planning became more concrete the problems became more evident.*
**Italy**	*I’ve been dating with my girlfriend since December. She is always on time and obsessed with punctuality, while I’m always late. After a while she has understood and realized I didn’t make it on purpose.*
**The Netherlands**	*I’m very happy with my wife. […] She writes everything down and she divides the tasks that have to be done. Because if I have to do that, nothing will come of it.*
**United Kingdom**	*I struggled a lot with thinking that there was something fundamentally wrong with me. […] Two years is all I can take, of anybody, and relationships seemed to break up. I was married for eight years, but the only way I was ever going to be married for eight years was because I was fooling around all the time.*
**United States**	*It drives my wife crazy because she is so bottom line and things need to make sense. I come out of left field with something crazy.*

**Table 20:** Problems with Friends/Meeting New People

	**Problems with Friends/Meeting New People**
	**Approximately 47 participants reporting (44%) with 42 of these (89%) noting difficulties with friendship and meeting new people**
**Canada**	*My friends say I can’t listen, or they say I can never listen to what they’re saying and I’m always talking about myself and nobody else. […] I do care about my friends, but I think a lot of the time they are right. It’s just so hard to listen to what other people have to say.*
**France**	*I am, by nature, very interested in things so whatever the job or the subject it interests me. I am quite an open person so it is not hard to get to know somebody, just afterwards it gets a bit more difficult as I am not ‘normal’ like other people; a bit strange, a bit bizarre. Therefore people think again.*
**Germany**	*So inattentiveness is important when you communicate with others, when you go out with friends. […] So it is important in communication, in your behavior with others, whether you are able to listen or whether you can communicate in a conversation.*
**Italy**	*Other things I feel very much: lack of awareness. Going back to my past, thinking about what I did, lack of awareness is a very bad thing. My life passed before my eyes as a film. I couldn’t get into it. It’s a matter of lack of awareness and I still have it now.*
**The Netherlands**	*No, I have a lot of acquaintances, but real friends, there aren’t many of those.*
**United Kingdom**	*People just don’t really understand me. […] On the positive side, they really like it when I’m feeling a bit up. I’ve got a lot of energy and […] they think I’m really great, […] and other times they just can’t believe just how depressed I am. It’s like, oh my god, stay away from her sort of thing, so it’s very difficult. I think people find that inconsistency difficult to deal with.*
**United States**	*It really bothers me that I won’t remember names.*

#### Self-image and self-esteem

The association of ADHD with behavioral traits accentuated participants’ reflections upon their self or person. Participants spoke of feeling different from others throughout life, regardless of country of origin, and they often spoke of having felt “not normal” or “wrong” as a result of this difference. They also spoke about positive personality attributes that they associated directly with having ADHD (Table 21).

**Table 21:** Self Image/Self-Esteem

	**Self Image/Self-Esteem**
	**Approximately 98 participants reporting (91%) with 75 of these (77%) noting problems with self image and self esteem**
**Canada**	*Well, I never took any medication, always full of complexes and in my own bubble, and full of anxiety, and if I have a party what will I do, what will I wear, and everything always became so complicated, and then at the last minute I’d look at myself in the mirror and say, “Hey, I’m not that ugly!” and I’d get to the party and, oh, boy, like, all the women were beautiful except me, you know, and I felt like a loser, for everything. And even now it’s better but I still have these complexes and low self-esteem, and I have to program myself in advance. And my boyfriend says, “Just live in the moment,” but I can’t, I’m not there, I’m over there. I’m not here, I’m there. Don’t ask me to be late and not ready, I have to be ready. And, you know, he’s really active, into sports and stuff, and I’m smooth but inside it’s all the anxiety. And I’m somewhere else in my mind, very often. It’s so weird.*
**France**	*It’s true that I notice after a while, you end up, how do you put it… I don’t see ADHD as an illness, but as a kind of specific feature. I don’t feel sick. I think my self-esteem swings. I have good days when I feel punchy, motivated, like a go-getter. And then an hour later, I’m trying to write my book and I tell myself, “Poor jerk, who’s going to read you? Everything you’re writing down has been said a thousand times. What have you done that’s innovative?” […] I feel inferior to other people. On the hand, sometimes, I feel superior in certain ways at least. I say, “You’d have to be a real idiot to have a nine-to-five Monday to Friday job, you’d have to be real jerk to have a boss, to get paid once a month. […] At the same time, I have a great amount of contempt for working people, people with a job. I want to say, liberate yourselves! You all have blinders on. In those instances, I feel like somebody who’s understood the world. And I see people all following the trodden path.*
**Germany**	*One aspect I’d like to mention that is the self-esteem problem. It is a great handicap I think. Although I’ve really made great inroads and I don’t see everything negative anymore but you sometimes get feedback from others that something isn’t as it should be and then you start to interpret and you start to over emphasize and you start to read too much into it.*
**Italy**	*Being loquacious is one of our characteristics. Before the diagnosis, the biggest problem I had and that heavily penalized me in my social life was my impulsiveness: I always fidgeted, I didn’t have self-control because I was not aware. Since I am aware I am ADHD (and I say I am ADHD, not “I have the ADHD”, I really care about this semantic differentiation!), the only awareness associated with the study of the pathology, the reading of books and comparisons with other people, helped me very much keep impulsiveness under control. So, my main symptom was impulsiveness, now it’s much better. I’m still impulsive, I wouldn’t define myself a quiet person.*
**The Netherlands**	*I used to finish hardly anything, but not anymore. At a certain moment you just have to, otherwise the problems will simply pile up. You will sooner or later run into the things you haven’t finished and get the consequences back from it. At first you laugh about it and think “that’s just who I am”. But if you keep on not finishing things, you don’t have a completed education, or you won’t stick to your appointments and get only negativity back because of that. So in the end you have to train yourself, with or without medication, so you won’t get that misery.*
**United Kingdom**	*I spent years psycho-analyzing myself on failed relationships and put a lot of blame on me, and a very good friend once said to me like to know me is to love me, and I thought what the hell? You know, if people don’t take the time to get to know you how’s anyone ever going to love you? But, the people that take the time out to get to know me are the people that are worth having in my life, and I just think I’m me and I like me now.*
**United States**	*I feel like there’s a lot of cognitive dissonance. You know that people are able to make routines and schedules and do things, but you can’t do it. So you’re setting yourself up, like: why can’t I do that? I don’t know why I can’t do that. Why can’t I? I feel like it reflects—I’ll just speak for me. I really have to work on addressing the issue rather than telling myself: you suck because you can’t do these things that obviously have a negative detriment on your life.*

### “Curing” ADHD

In each focus group, participants were asked whether or not they would have their ADHD instantly “cured” or “taken away” if this were possible. Participants’ responses to this question ranged the entire spectrum from absolutely no to yes. Many respondents displayed ambivalence in their response, and they talked about ADHD being an important part of their personality structure and that it included both positive and negative qualities. In these focus groups and interviews, 30 (28%) reported that they would agree to having ADHD removed from their lives, 38 (35%) said that they would not agree, and 17 (16%) were ambivalent on the issue. The remaining 23 participants (21%) did not respond to the question (Tables 22 and 23).

**Table 22:** Removing or Curing ADHD

	**Removing or Curing ADHD**
	**Approximately 85 participants reporting (79%)**
**Canada**	*It might be good for kids, this magic wand, so they can evolve normally, because as an adult now I’ve learned to live with it, I’ve evolved with it, and here this is where I am because I have had my ADHD for virtually ever, so it’s like even if… okay, it affected my life, you know, two months, six months, a year ago, fine, but I can’t really say it was affecting it that differently. It’s hard to explain, because I am the way I am and I was always like this. I’m a result of all this, so how can I tell you is it because I had ADHD that that affected whatever part of my life, or is it because things happened like that? So I don’t know, it’s hard to explain.*
**France**	*That’s what makes us different from people with different types of personalities, people who are so-called normal. But I want to take it away because of the suffering, the pain it brings. When something bad happens, I have a physical sensation of feeling bad. I feel nervous. I feel like doing something but I feel like something is hindering me. When I was diagnosed with ADHD, it gave me the feeling that I had a user manual, a road map. Now I know more about what’s the matter with me. I feel more serene. Now I know where my problems come from. I don’t control it yet. I’m hoping the diagnosis will give me better control over myself.*
**Germany**	*I don’t know, I’m not quite sure. My brother doesn’t have it and he leads a pretty good life I think. And sometimes I’d like to live my life like he does. I’m a little creative as well but I haven’t turned it into my job, so I cannot enjoy it as much. I probably would be glad if I didn’t have it but if I didn’t have it I don’t know what life would be like. Perhaps life would be more boring.*
**Italy**	*Positive I don’t think. I’d rather not have ADHD sincerely. I think the only positive aspect is that whatever I have had, no matter what happened to me, led me to be the person I am now and I’m happy. Now I’m good like this. […] I said if I might not have it, I’d be better. It’s a dysfunction. […] Apart from saying that now I’m fine with it. I’m one of those who say “Now it’s very good. It could be even better”. If there was the magic pill, I’d absolutely say yes…*
**The Netherlands**	*Yes, I was. Only it’s just… It’s guessing. I’ve had a fine life, I’ve done everything I wanted, it may seem a bit strange to normal people but I wouldn’t want to do anything different.*
**United Kingdom**	*I don’t know, I think it’s probably done good for me, for the first part of my life, certain things I was doing. But now, I don’t think I need it anymore, if you know what I mean? I don’t need that to be alert and aware as I have been previously. So, I don’t know, perhaps I need a bit of a break from it. I’d like it gone.*
**United States**	*My family is really dysfunctional. And my entire life I’ve always just gone through like: oh, well—and really accepting. And I think it’s because with the ADHD I always find something else to replace the negative. Like, oh, I lost a finger—oh, I still have nine more. I’ve never gotten depressed. I’ve never had bad thoughts or anything like that. I’ve always been able to balance it out. And I think if I didn’t have ADHD—the kind of life that I’ve lived is really terrible, you know. I’d really rather not talk about it because I’m not looking for pity or anything like that, but it’s just kept me really happy and really accepting for anything that happens. So that’s a really great side to it.*

**Table 23:** Positive Aspects of ADHD

	**Positive Aspects of ADHD**
	**Approximately 49 participants reporting (45%)**
**Canada**	*I don’t know. You get a lot done, you know? So my life is really fulfilling. I don’t know. I think the way my life went was more traumatizing than the condition.*
**France**	*Maybe it sounds strange, but for me, impulsiveness is almost a good quality. If you’re impulsive, it means that you say what you do and do what you say. You’re more natural. You’re not trying to be Mr. Everyman, you’re not a sheep in the herd. You’re imposing your point of view.*
**Germany**	*I think I can do much more work with my condition and who knows and if I’m out and about then it is good that I’ve got this extra energy this overabundance of energy if it is not about fine details and fine tuning.*
**Italy**	*It’s not true. We know what we like. Maybe you are not ADHD and you like going cycling, another one may like skiing. We have this individuality as well. And if we like something we are very good, not good, very good. If we don’t like doing something, it’s really hard. In order to do something well, ADHD people don’t need a motivation, but a supermotivation, a further step rather than motivation. When there’s a supermotivation we are exceptional.*
**The Netherlands**	*Yes, I would. I’ve just experienced so many weird and fun things, and you wouldn’t have if you don’t have it. It’s not like a life without ADHD is more boring by definition, but I know for sure that your life can be a lot more fun with ADHD.*
**United Kingdom**	*Yes, you’re sparky, being fearless, just doing really crazy stuff sometimes, not feeling inhibitions. In the positive sense, not the negative sense, and being able to be creative and see through things and you know, laugh about stuff normal people probably, well people whatever, who wouldn’t usually laugh about, just silly, quirky little things.*
**United States**	*But I wouldn’t give away the creative part, where I can see outside of the box. I enjoy that. I enjoy my interest in different things. I enjoy being able to see different possibilities that other people don’t even think of, the creative aspect.*

## Discussion

These findings strongly suggest that, on an international scale, the experience of ADHD in daily life is consistent, despite the socio-cultural differences that may exist between these seven countries. These adults experienced the impacts of ADHD on functioning and well being similarly across national boundaries. Participants in these focus groups felt themselves to be different from others, often from childhood, and reported that receiving a diagnosis of ADHD as adults can be accompanied by substantial difficulty. They retrospectively identifed difficulty interacting with others in childhood, and noted a great deal of difficulty in school life and with the educational process. They also believed that ADHD has impacted their lives in very significant ways, especially with regard to education and career, and their statements support clinical research conclusions that people with ADHD are prone to troubles with addictive behaviors and other activities thought to be risky or dangerous. Navigating daily life presented considerable challenges for these adults as they reported difficulties with social relationships of all types, including with work colleagues, family, friends, and acquaintances. Together, these challenges appear to contribute to psychological conflict and diminished well-being for many. Given that 42% of these adults reported other mental health conditions along with their ADHD, some caution is suggested in attributing all impairments reported solely to their ADHD. However, in general, the statements of subjects who reported other mental health conditions and the greater majority (58%) who did not report other conditions were not substantively different.

Furthermore, these adults agreed on the key presentation of symptoms of ADHD: hyperactivity, impulsivity, inattention, and disorganization without regard to their cultural and social biases. It may not be surprising that people who have been diagnosed with a condition, presumably in accordance to standardized symptom criteria, might agree on the symptoms most central to the condition itself. This is the situational background of this study, which recruited participants who had received a diagnosis of ADHD into focus groups to explore ADHD. This is also this study’s major limitation. Similarly, the fact that participants in these focus groups retrospectively identified problems in childhood also resonate with the explicit diagnostic criteria that a retrospective assessment of childhood is included in the diagnostic process. However, despite the standardized criteria of biomedicine, one might surmise that a condition that centers on observable behaviors might be experienced differently across national and cultural borders, resulting in different impairments in daily life and lifespan activities due to interpretation bias by society or culture. However, these focus groups suggest that this is not the case for these seven countries and that the burden of illness is, instead, quite similar for adults with ADHD.

Limitations for this small study include a self-selection bias by the participants. As interested volunteers for the study, they may not have been an adequate representational sample for the entire national populations of adults with ADHD. In addition, the study’s focus on the burdens of ADHD may not have captured fully the perceptions these individuals may share with regard to its positive qualities. The emphasis on illness in the focus group script was concomitant with an emphasis on debility, rather than ability. Furthermore, these methodological limitations point to the need to confirm the results quantitatively.

It should be noted that not all countries participating in this study have a medical system that relies on similar diagnostic categories, general criteria for diagnosis or the availability of ADHD medications for adults. For example, in Italy and France, ADHD is recognized primarily as a condition of childhood and commonly used medications for ADHD are not prescribed or generally available for adults. Thus the participants in the focus groups held in Italy spoke of the need to travel to other countries for both diagnosis and medicines, thus making their diagnosis experience and treatment somewhat more challenging and emotionally difficult than those of individuals in the United States, Canada, or the United Kingdom. Relatedly, health systems differences, while not explored to a great extent in these focus groups, may be the more salient difference across national boundaries than the diagnosis and experience of ADHD. In a world with global access to biomedical information and related media, coupled with international diagnostic guidelines, differences between countries may be less pronounced.

An important finding of this study centers on medication use. Among those who are prescribed and use medication for their ADHD symptoms, there appeared to be general satisfaction that the medications were efficacious and helpful to them. However, these individuals generally did not take their medications in compliance with their prescriptions, but as they saw fit based upon their interpretation of their signs and symptoms. This opens up a potential area of investigation into the impacts of adherence and non-adherence to medications for ADHD and its effects on long-term burden of illness. Further study investigating the effects of intermittent and fluctuating use of medication for ADHD symptoms in adults, conducted through both qualitative and quantitative methodologies, may lend more insight into the complexities of adult ADHD treatment. Furthermore, although not definitive, the commentaries of these respondents identified that side effects, perceived by some to be deleterious, led to a refusal of the medication itself. These patient-reported outcomes should be considered in the development of future treatment modalities, whether these treatments are pharmaceutical or behavioral.

Findings from this study suggest that additional qualitative research is needed to further elucidate socio-cultural differences across countries generally with regard to ADHD in order to better understand the context within which people experience their disease. This ethnographic research might include expert interviews with clinicians from each country, including those whose knowledge centers on ADHD and those whose practice may be more general, as well as attitudes from the lay public who do not have a diagnosis of ADHD. Parsing the role of social stigma from the symptoms themselves could be used to understand how social experiences impact the burden of illness in the case of ADHD. Focus groups segregated by the time of diagnosis (childhood or adulthood) and across multiple countries would provide additional insight into a very intriguing area of research.

In addition to ethnographic investigation, there are many other intriguing questions associated with ADHD in adults, clinical recognition, and health system contributions to the burden of illness. These include the levels of knowledge and acceptance of ADHD in adults among potentially treating practitioners, the accessibility of medical intervention and services within specific health systems, and the availability of multiple treatment medications. Within patient experience, it would also be interesting to be able to understand 1.) the relationship between the numbers of years diagnosed with ADHD and burden of illness; 2.) co-morbid mental health conditions and adherence to treatments; and 3.) feelings of social isolation and burden of illness.

Last, all of the countries selected for this study are considered “western” countries. As such, each has similar social and economic structures for education, work, and social relationships. It is not surprising, therefore, that participants in this study might have similar life stories to tell about the obstacles they faced as people with a diagnosis of ADHD. What is of importance, however, is the extent to which adults with a diagnosis of ADHD agree upon the burden of illness and general life impacts. Similar qualitative investigation into the burden of illness of ADHD in adults that includes countries outside North America and Europe would be an important contribution to this field.

## Conclusions

ADHD is a consistent and stable diagnostic category across North America and Europe among those who carry the diagnostic label as adults. This appears to be true even in countries in which doctors typically do not recognize ADHD among adults. As these data demonstrate, adults in the United States, Canada, United Kingdom, France, Germany, The Netherlands, and Italy shared similar narratives in primary domains of functioning and well-being and agreed on the fundamental symptoms of ADHD. This suggests that the burden of illness and impacts of ADHD are similar across these seven countries and not limited to the United States alone.

## Abbreviations

ADHD: Attention Deficit-Hyperactivity Disorder.

## Competing interests

Meryl Brod, PhD and Betsy Pohlman, PhD are paid consultants to Shire Pharmaceuticals and do not own stock in the company. Robert Lasser, MD and Paul Hodgkins, PhD are paid employees of Shire Pharmaceuticals and own stock in the company.

## Authors’ contributions

MB participated in study design, conducted focus groups, and participated in preparation of manuscript. BP conducted analysis of data and participated in preparation of manuscript. RL and PH participated in study design and preparation of manuscript. All authors read and approved the final manuscript.
